# Supplementary material for: Impact of genomic literacy components on genetic testing decision-making in the general Japanese population in the 20s and 30s
Source: PLoS One. 2023 Mar 29;18(3):e0283432. doi: 10.1371/journal.pone.0283432 (PMC10057763; doi:10.1371/journal.pone.0283432)
Supplement: Attachment — Submitted filename: Response to Reviewers.docx [file pone.0283432.s002.docx]

Dr. Qasim Ayub

Academic Editor

*PLOS ONE*

U.S. Headquarters Public Library of Science

1160 Battery Street, Koshland Building East, Suite 100

San Francisco, CA 94111 United States

2^th^ March 2023

Re: Manuscript ID: PONE-D-22-23128

Dear Dr. Ayub,

Thank you very much for your e-mail and review of the manuscript (PONE-D-22-23128) that we sent on 20^th^ August 2022. We sincerely thank the two reviewers for providing constructive comments regarding the improvement of the original manuscript. Thus, it is with great pleasure that we resubmit our article for further consideration. We have incorporated changes that reflect the detailed suggestions you have graciously provided. We also hope that our edits and the responses we provide below satisfactorily address all the issues and concerns you and the reviewers have noted. To facilitate your review of our revisions, the following is a point-by-point response to the questions and comments delivered in your letter dated 23^rd^ December 2022.

Editor's Comment:

**Please ensure that the data associated with the manuscript or its supporting information are available via a public repository following PLoS One guidelines and update the methodology section to address the concerns raised by Reviewer #1. In addition, please update Figure 1 and Table 1 and check that all references are complete and correct.**

RESPONSE: Thank you for taking the time to provide us with your valuable feedback.

**Additional Information:**

**Financial Disclosure**

RESPONSE: The authors received no specific funding for this work.

**Competing Interests**

RESPONSE: The authors have declared that no competing interests exist.

**Ethics Statement**

RESPONSE: We created a section on "Ethical Considerations" and added content as follows:

The research company that conducted this survey has obtained the Privacy Mark and ISO/IEC27001 (ISMS) certifications from the Japan Information Processing Development Corporation (JIPDEC), and is an organization that gives due consideration to the protection of registered users' personal information, and has obtained the Privacy Mark and ISO/IEC27001 (ISMS) certifications. The personal information of the monitors is managed by the research company, and no more personal information than necessary will be obtained by the researcher. This study has been approved by the University's Research Ethics Committee (approval number: 449). The purpose of the study and the handling of personal information were explained on the webpage, and responses were considered to be the acquisition of consent.

**Data Availability**

RESPONSE: The data that support the findings of this study are openly available at: https://figshare.com/, reference number (10.6084/m9.figshare.22122380).

Reviewer #1 comments.

RESPONSE: Thank you for taking the time to provide us with your valuable feedback. Our response to the referee's comments is as follows.

**Page 4: Better to cite some related studies in Japan and then bring up the research gap.**

RESPONSE: We thank the reviewer for this comment. We have added the following statement in accordance with the reviewer's comment:

“In Japan, decision-making research on genetic testing is limited to the perception of patients

undergoing genetic testing and their decision support.” (lines 97–98)

**Page 5: The authors need to mention how they calculated the sample size and the sampling technique used to draw this sample.**

RESPONSE: We thank the reviewer for this comment. In accordance with the reviewer's comment, we added the following statements:

“The survey covered 2,000 respondents (1016 males and 984 females) between the ages of 20 and 59, and 857 respondents (438 males and 419 females) between the ages of 20 and 39 were included in the analysis of this survey. Since it has been noted that online surveys can bias respondents based on factors such as gender and age, the number of respondents for this survey was determined after evenly stratifying the gender and age groups according to the population distribution and percentages in the 2019 national survey.” (lines 109–114)

**Page 8: There is no information about testing correlation and regression analysis assumptions. Authors must provide this information.**

RESPONSE: We thank the reviewer for this comment. In accordance with the reviewer's comment, we added the following statements:

“Next, a correlation analysis was conducted to examine the relationship between the three genomic literacy variables, risk perception score, benefit perception score, and behavioral intentions toward genetic testing. To investigate the effects of genomic literacy components on behavioral intention toward genetic testing, those who had a genetic disorder or a family member with a genetic disorder were excluded and a multiple regression analysis was performed, with behavioral intention towards genetic testing as the dependent variable and the three components of genomic literacy as the independent variables.” (lines 196-202)

**Page 11: What is the p-value here?**

RESPONSE: Thank you for pointing this out. Accordingly, we have added the p-value to this section within line 234 of the revised manuscript.

**Page 14: It would be better if the authors provided a few subtitles for this section and then discussed the individual objectives under each subtitle.**

RESPONSE: We thank the reviewer for this comment. In accordance with the reviewer's comments, we have added subtitles and discussed the individual objectives. We have made additions to the wording of the manuscript to lines 302-303 as follows:

“Relationship between genomic literacy components, risk perception, benefit perception, and behavioral intention toward genetic testing” and to lines 326-327 as follows:

“Impact of genomic literacy on behavioral intentions via risk perception and benefit perception” in the revised manuscript.

Reviewer #2 comments.

Comments on the overall

**Fig 1 is referred to in the text. Unfortunately, the Fig was presented in the manuscript. This made it difficult to follow and evaluate all aspects of the manuscript that made reference to it. Overall the manuscript is in good shape. The methodology is robust and the statistical analysis is sound. However, there are a few minor errors in the manuscript. Some of these are shown below:**

RESPONSE: Thank you for taking the time to provide us with your valuable feedback, and we apologize for the same.

**Line 78-19 Please provide references pertaining to the many authors.**

RESPONSE: In accordance with the reviewer's comment, we have now added all the cited references.

**Line 117 to read** **"Questionnaire items"**.

RESPONSE: We thank the reviewer for this comment. In accordance with the reviewer's comment, we have changed this to "Questionnaire items."

**Line 213 Table 2 does not show any % contrary to the claim made in line 213. The authors should attend to the formatting of the table so that it reflects all the data. Although the percentages are mentioned in the text, it is important to make sure that the Table is complete.**

RESPONSE: We wish to thank the reviewer for this comment. In accordance with the reviewer's comments, we added an "incorrect" and "don't know" entry to Table 2.

Once again, we thank you for giving us the opportunity to strengthen our manuscript with your valuable comments and queries. We have worked hard to incorporate your feedback and hope that these revisions persuade you to accept our submission.

Sincerely,

Miyoshi Tomomi

Department of Cardiovascular Medicine, The University of Tokyo Hospital, Tokyo, Japan.

E-mail: miyoshi.tomomi@mail.u-tokyo.ac.jp

Masaki Watanabe

Graduate School of Teacher Education, Tokyo Gakugei University, Tokyo, Japan.

E-mail: masawata@u-gakugei.ac.jp
